# Identification of Data Injection Attacks in Networked Control Systems Using Noise Impulse Integration †

**DOI:** 10.3390/s20030792

**Published:** 2020-01-31

**Authors:** Alan Oliveira de Sá, António Casimiro, Raphael C. S. Machado, Luiz F. R. da C. Carmo

**Affiliations:** 1 Admiral Wandenkolk Instruction Center, Brazilian Navy, Rio de Janeiro RJ 20180-003, Brazil; 2 Institute of Mathematics/NCE, Federal University of Rio de Janeiro, Rio de Janeiro RJ 21945-970, Brazil; lfrust@inmetro.gov.br; 3 Department of Informatics, Faculty of Sciences of the University of Lisboa, 1749-016 Lisboa, Portugal; casim@ciencias.ulisboa.pt; 4 National Institute of Metrology, Quality and Technology, Xerém RJ 25250-020, Brazil; rcmachado@inmetro.gov.br; 5 Institute of Computing, Fluminense Federal University, Niterói RJ 24210-310, Brazil

**Keywords:** security, industrial control system, networked control system, data injection attack, countermeasure, system identification

## Abstract

The benefits of using Networked Control Systems (NCS) in the growing Industry 4.0 are numerous, including better management and operational capabilities, as well as costs reduction. However, despite these benefits, the use of NCSs can also expose physical plants to new threats originated in the cyber domain—such as data injection attacks in NCS links through which sensors and controllers transmit signals. In this sense, this work proposes a link monitoring strategy to identify linear time-invariant (LTI) functions executed during controlled data injection attacks by a Man-in-the-Middle hosted in an NCS link. The countermeasure is based on a bioinspired metaheuristic, called Backtracking Search Optimization Algorithm (BSA), and uses white Gaussian noise to excite the attack function. To increase the accuracy of this countermeasure, it is proposed the Noise Impulse Integration (NII) technique, which is developed using the radar pulse integration technique as inspiration. The results demonstrate that the proposed countermeasure is able to accurately identify LTI attack functions, here executed to impair measurements transmitted by the plant sensor, without interfering with the NCS behavior when the system is in its normal operation. Moreover, the results indicate that the NII technique can increase the accuracy of the attack identification.

## 1. Introduction

**Figure 1:**
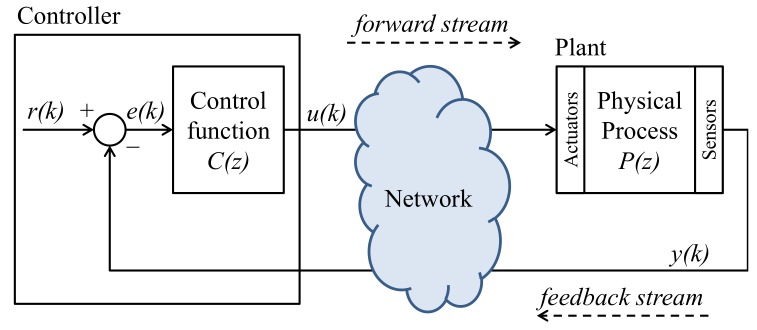
Networked Control Systems (NCS) [8].

The concept of the fourth industrial revolution—Industry 4.0 [1,2]—arises with the development and use of cyber-physical systems, which promote the computerization of manufacturing and integrate communication networks to physical processes. In this scenario, Networked Control Systems (NCS)—i.e., controllers and sensors/actuators of physical plants connected through communication networks [3,4,5,6,7]—are widely used to obtain better management and operational capabilities, as well as cost reductions [8]. In an NCS, as shown in Figure 1, a controller—i.e., a computer system—executes a control function C(z) to properly drive the behavior of a physical plant, herein described by a discrete-time transfer function P(z). The control signal produced by the controller is transmitted to the plant actuators through a forward stream. The signals measured by the plant sensors, in turn, are sent to the controller through a feedback stream.

The possible applications for NCSs are broad and can range from non-critical industrial plants controlled by wireless networked control systems (WNCS) [9], to critical infrastructures controlled by wired NCSs, such as nuclear reactors [6,10,11] and water canal systems [12]. However, despite the several benefits provided by NCSs, the use of communication networks to integrate controllers and physical plants can also expose these systems to cyber threats [8,12,13,14,15,16]. Indeed, the literature [7] reports the execution of real cyber-attacks against physical plants since 1982, affecting a wide variety of targets, such as a diesel generator, a gas pipeline, and a steel plant. Among these known cases, the most emblematic example of attack in a cyber-physical system is the Stuxnet worm [14], whose targets were uranium enrichment centrifuges in Iran [17]. To achieve its aim, Stuxnet installed a modified control algorithm into the controller (a programmable logic controller—PLC) in order to cause subtle and harmful behaviors to the centrifuges, reducing their efficiency and causing damage [14,17,18].

Please note that one possible way to attack an NCS, for example, is by hacking its software (i.e., changing the configuration or even the code executed by the controller), following a strategy similar to that used by the Stuxnet worm [14]. Another possible way for an attacker to negatively affect an NCS is by interfering on its communication process between controllers, sensors and actuators. Basically, an attacker may interfere in the forward and/or feedback streams by three different means: inducing jitter, causing data loss due to packet drop outs, or even injecting false data in the communication process due to failure or absence of security mechanisms in the NCS.

In fact, although some new industrial communication protocols were developed including security features [9,19,20], there are protocols in industry that still lack security mechanisms [21]—such as the Profinet, MODBUS/TCP, and Ethernet/IP. The main issue of these industrial protocols is the lack of encryption and authentication [21] between devices (e.g., controllers, actuators, and sensors) used in automation and control systems. A vast collection of scientific literature about cybersecurity in Industrial Control Systems (ICS) is available, reporting security breaches in all major Real-Time Ethernet (RTE) protocols used in industry [21,22,23,24,25,26,27,28]. Therefore, considering the feasibility of occurring cyber-attacks against physical systems, as demonstrated by the real cases already reported in the literature [7,14,17], studies have been conducted aiming to characterize vulnerabilities and promote security solutions for NCSs [8,12,13,15,16,29].

In [12,15], it is proposed a covert misappropriation attack, where a malicious agent uses the knowledge about the plant model to inject false data in the NCS. The author assumes that the attacker knows the plant model, but does not describe how the model is obtained. More recent works [8,13] demonstrate that Service Degradation (SD)-Controlled Data Injection attacks can be accurately built based on the NCS models previously learned through system identification attacks [8,13]. The harmful effects that SD-Controlled Data Injection attacks can produce on physical plants motivate the research on mechanisms able to prevent them, as well as to detect/identify them when they occur.

It is possible to verify in the literature [7,8,12,13,15,16,21,22,26,29,30,31,32,33,34,35,36] that in cyber-physical systems (which includes NCSs), a relevant portion of the attack surface often lies in the communication process between sensors/actuators and controllers. For this reason, in the ICS cybersecurity landscape, significant attention has been given to the study of cyber-attacks to sensor/actuator systems [7]. Indeed, the high accuracy desired for sensors, for instance, may be useless if the integrity of sensor data is compromised by some kind of malicious manipulation in its communication process. Not by chance, still taking the scope of sensors as an example, data integrity is arising as a property as important as other typical sensor properties—e.g., accuracy, sensitivity, linearity, resolution, repeatability, etc. [32,34,35,36,37,38,39,40].

Aiming to improve the cybersecurity of NCSs, the authors of [29] discuss countermeasures that can be used to mitigate data injection attacks executed within the communication between sensors/actuators and controllers. These countermeasures can be systematically thought in a layered defense strategy [29] to avoid access to the control loop and data. Non-authorized access to the NCS control loop can be obtained, for instance, by using network segmentation, demilitarized zones (DMZ), firewall policies and implementing specific network architectures, such as described in [31]. Additionally, non-authorized access to data transmitted by controllers and sensors can be obtained by using security mechanisms for data confidentiality, integrity and authenticity. Such a solution is presented in [32], where the authors propose a countermeasure that integrates a symmetric-key encryption algorithm, a hash algorithm and a timestamp strategy to form a secure transmission mechanism between the controller side and sensors/actuators located in the plant side. However, it is noteworthy that even when NCS uses secure communication protocols and network architectures, existing security mechanisms can still be overcome. The security of the communication between sensors, controllers and actuators may be compromised, for instance, if an attacker succeed in obtaining security keys or passwords (used for encryption and authentication) through social engineering attacks [41]. In this case, as shown in [8,13], an attacker can have the conditions required to implement an SD-Controlled Data Injection attack. Therefore, it is important to develop countermeasures able to detect and identify SD-Controlled Data Injection attacks in NCSs.

In this sense, this work proposes a link monitoring strategy to identify linear time-invariant (LTI) transfer functions performed by a Man-in-the-Middle (MitM) during an SD-Controlled Data Injection attack [8]. The proposed countermeasure uses white gaussian noise to excite possible attack functions in the NCS, to obtain the information necessary to identify the attack. Moreover, to increase the accuracy of the attack function identification using white gaussian noise, this work also proposes a Noise Impulse Integration (NII) technique, which is developed inspired by the pulse integration process of radar systems [42]. From the NCS owner perspective, the knowledge about the attack function may be useful, for instance, to:

- provide information for an autonomous process intended to redesign the NCS control function, to mitigate the attack effects in the plant behavior;
- reveal the attacker intentions, for forensic purposes, helping to estimate the possible impacts of the attack on the plant and its services.

Previous works [37,38,39] report the use of Independent and Identically Distributed (IID) noise sequence as watermark to detect data injection attacks (integrity attacks) in NCSs. More specifically, the solutions proposed in [37,38,39] provide a physical authentication scheme to detect replay attacks in sensors’ measurements when the NCS is in steady state. In [38,39], the core idea of the detection scheme is to add an IID noise to the control signal applied to the plant and, thus, obtain a physical watermark within the plant output signal—transmitted by sensors—in a system equipped with a $\chi^{2}$ failure detector. In [37], to detect counterfeit sensor signals, the authors investigate the problem of designing the optimal watermark signal in the class of stationary Gaussian processes. Their results generalize the solution proposed in [38,39] where only IID Gaussian processes are considered in the design of watermarked control inputs. Also, the authors propose a watermark design method that bounds the control performance loss incurred by the watermark signal—note that although the cost to control performance is bounded, it is not completely eliminated. As mentioned by the authors, this drawback occurs in all solutions presented in [37,38,39]. The presence of the extra watermark signal in the control signal causes the control performance to not be optimal—i.e., to allow the attack detection, the control performance is sacrificed. In [40], the authors propose a multiplicative watermarking scheme to detect and isolate replay attacks on sensors measurements without interfering in the control performance. Unlike [37,38,39], to avoid detrimental effects on the closed loop performance, each sensor output is separately watermarked while an equalization filter is incorporated at the controller’s side to reconstruct the original plant outputs.

In the present work, differently from [37,38,39], the proposed solution is designed not to sacrifice the system performance when it is in normal operation. Here, the white gaussian noise added in the transmitting device (e.g., a sensor) is cancelled in the receiving device (e.g., a controller), in a strategy analogous to the multiplicative watermarking scheme used in [40]. Moreover, while the watermarking schemes proposed in [37,38,39,40] aim to detect data injection attacks (specifically, replay attacks) in sensors measurements, they do not intend to identify possible LTI attack functions within the communication between sensors/actuators and controllers. In the present work, differently from [37,38,39,40], the proposed solution is intended to detect and identify (i.e., estimate the parameters of) LTI attack functions executed during data injection attacks in NCSs—precisely the class of SD-Controlled Data Injection attacks discussed in [8,13]. The identification of SD-Controlled Data Loss attacks using switching LTI attack functions is not considered in this paper.

It is worth mentioning that the proposed countermeasure is not intended to prevent the implementation of an SD-Controlled Data Injection attack, but to detect and identify it once it occurs, in order to obtain knowledge about the attack function. As in other works addressing the detection of data integrity attacks in NCS [37,38,39], the countermeasure herein proposed is designed to identify the attack when the plant is operating in steady-state condition – which is still a relevant system condition to be considered in cybersecurity of NCSs [37,38,39]. Please note that SD attacks, by definition [8], are not intended to cause immediate system failure. They are intended to degrade the efficiency of the physical process or to reduce the mean time between failure of the plant, remaining active in the system for mid/long term. Thus, the knowledge about the attack function obtained during steady operating conditions is useful to build reactive countermeasures that make the attack cease (or mitigate it) once it has started—even if the beginning of the attack was during a transient response and its identification occurs during the subsequent steady condition.

The reminder of this work is organized as follows: Section 2 briefly presents the concepts of the SD-Controlled Data Injection attack [8]. Section 3 describes the proposed countermeasure—a link monitoring mechanism—including the NII technique herein introduced to increase the accuracy of the attack identification. Section 4 shows simulation results that evaluate the performance of the proposed countermeasure when identifying an SD-Controlled Data Injection attack in the communication between a sensor and a controller. It also evaluates the ability of the NII technique in increasing the accuracy of the identification process. Finally, Section 5 brings the conclusions of this work.

## 2. SD-Controlled Data Injection Attack

For the sake of completeness, this section briefly describes the SD-Controlled Data Injection attack characterized in [8]. The attack purpose is to reduce the mean time between failure (MTBF) of the plant and/or reduce the efficiency of the physical process that the plant performs, by inserting false data in the NCS communication links.

**Figure 2:**
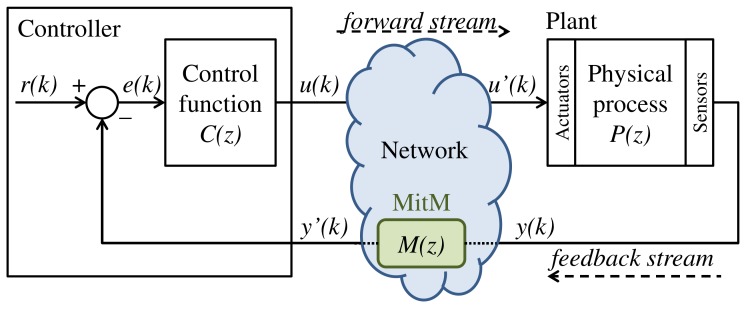
SD-controlled data injection attack.

In the SD-Controlled Data Injection attack, to cause a harmful behavior on the plant (e.g., an overshoot or a steady-state error), the attacker interfere in the NCS’s links by injecting false data into the system in a controlled way. To do so, the attacker act as a MitM that executes an LTI attack function M(z) between the sensor and the controller, as presented in Figure 2, wherein $Y^{\prime }\left(z\right)=M\left(z\right)Y\left(z\right)$, $Y^{\prime }\left(z\right)=Z\left[y^{\prime }\left(k\right)\right]$, Y(z)=Z[y(k)], and Z represents de Z-transform operation. The function M(z) is designed based on the models of the plant and the controller, both obtained through a System Identification attack [8,13]. Therefore, according to [8], the SD-Controlled Data Injection attack is implemented in two subsequent stages:

STAGE-I: The system identification stage [8,13] is executed to provide the attacker an accurate knowledge about the models of the targeted system, i.e., the plant’s transfer function P(z) and the controller’s control function C(z). This knowledge is obtained either through a Passive System Identification process [8] or through an Active System Identification process [13].
STAGE-II: The Data Injection stage is performed. The attacker, as an MitM, injects false data in the NCS control loop. To accurately change the plant physical behavior, the injected false data is computed according to M(z) which, in turn, is designed based on the knowledge obtained by the attacker during STAGE-I.

## 3. Identification of Controlled Data Injection Attacks

This section proposes a countermeasure—a link monitoring strategy—to identify the LTI transfer function performed by a MitM during an SD-Controlled Data Injection attack (described in Section 2). Section 3.1 describes the proposed link monitoring strategy, which uses white gaussian noise to excite the attack function and obtain the information necessary for the identification process. The countermeasure is designed to do not affect the plant behavior in normal operating conditions (i.e., without attack). To estimate parameters of the LTI attack function, the identification process uses a bioinspired metaheuristic called Backtracking Search Optimization Algorithm (BSA) [43]. Additionally, to increase the accuracy of the attack identification using white gaussian noise, this work proposes a Noise Impulse Integration (NII) technique, which is presented in Section 3.2.

### 3.1. Strategy to Identify the Attack

**Figure 3:**
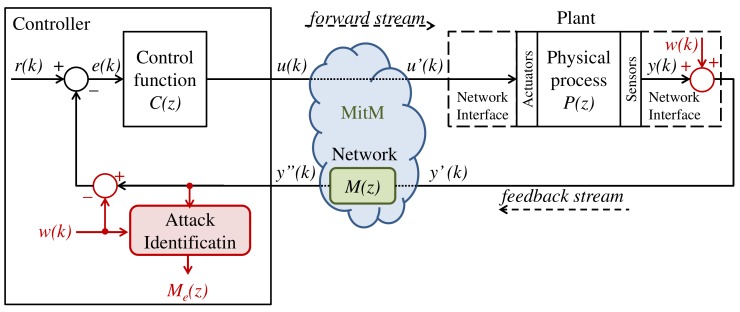
Identification of an SD-Controlled data injection attack [45].

This section describes a link monitoring strategy to identify the LTI attack functions used by a MitM during the SD-Controlled Data Injection attack defined in Section 2. Consider, for instance, the SD-Controlled Data Injection attack shown in Figure 3, where the attacker only has access to the measurements transmitted by the sensor to the controller through the feedback stream.

To identify the attack function, M(z) must be excited by an input signal to produce meaningful information for the identification process. If the system is in steady operating conditions, for instance, the information content of measured signals is often insufficient for identification purposes [44]. Considering this, one possible strategy to identify an attack function is to use typical variations in the NCS signals—such as a variation caused by a change in the setpoint r(k)—to estimate M(z). However, depending on the system, these variations may not occur often, which can make the identification of M(z) time consuming. Furthermore, causing arbitrary variations in such signals to identify M(z) may not be convenient as it may affect the behavior of the plant.

The architecture shown in Figure 3 is proposed as a solution that can be used to excite M(z) at any time, without affecting the plant behavior when the system is working in normal conditions—i.e., without attack. To do so, as shown in Figure 3, a white gaussian noise w(k) is injected (added) in the signal to be transmitted through the monitored link. To avoid interfering in the controlled plant when the system in not under attack, the same noise signal w(k) is subtracted from the monitored NCS signal at the other end of the link. In Figure 3, where the feedback link is the one being monitored, w(k) is injected at the sensor’s network interface, and subtracted at the controller input. In this system, the NCS output Y(z)=Z[y(k)] is defined as (1):

(1) $$Y\left(z\right)=\frac{C(z)P(z)}{1+C(z)P(z)M(z)}\left[R\left(z\right)+W\left(z\right)\left(1-M(z)\right)\right],$$

wherein R(z)=Z[r(k)] and W(z)=Z[w(k)]. Please note that if w(k) is exactly the same signal at both ends of the monitored link and the system is not under attack (i.e., M(z)=1), then the injection of w(k) is cancelled and does not influence y(k). In this case, based on (1), the plant output Y(z) is defined as (2):

(2) $$Y\left(z\right)=\frac{C(z)P(z)}{1+C(z)P(z)}R\left(z\right).$$

The white gaussian noise w(k) is chosen to excite the attack function due to its unpredictability, which makes it harder for an attacker to estimate the noise that will be added to the link at any given moment. The white gaussian noise w(k) is obtained from a normal distribution, such that w(k)∼N(μ,σ), wherein μ=0 is the mean and σ is the standard deviation. To have the same noise signal w(k) at both ends of the monitored link, it is considered that these two sources of noise are synchronized and both signals are produced based on the same seed. Moreover, to avoid an attacker to predict the noise values, the seed is exchanged among both devices—i.e., the transmitter and receiver—using a secure key exchange method, such as the Diffie-Hellman algorithm [46].

Now, if the system is under attack (i.e., M(z)≠1), then, according to (1), the noise is not cancelled. In this case, the signal observed at the controller input $y^{\prime \prime }\left(k\right)$ is given by (3):

(3) $$\begin{array}{c} y^{\prime \prime }\left(k\right)=\underset{y_{1}^{\prime \prime }\left(k\right)}{\underset{︸}{w\left(k\right)*Z^{-1}\left[M\left(z\right)\left(\frac{1+C(z)P(z)}{1+C(z)P(z)M(z)}\right)\right]}}+\\ \underset{y_{2}^{\prime \prime }\left(k\right)}{\underset{︸}{r\left(k\right)*Z^{-1}\left[\frac{C(z)P(z)M(z)}{1+C(z)P(z)M(z)}\right]}}. \end{array}$$

In the present countermeasure, the identification of M(z) is performed by observing the variations produced by w(k) in $y^{\prime \prime }\left(k\right)$ when M(z)≠1. Note, in Figure 3, that both w(k) and $y^{\prime \prime }\left(k\right)$ are provided to the Attack Identification process. The effect of w(k) in $y^{\prime \prime }\left(k\right)$ is specifically indicated in (3) as $y_{1}^{\prime \prime }\left(k\right)$. To have the identification relying on $y_{1}^{\prime \prime }\left(k\right)$, and independent from variations in $y_{2}^{\prime \prime }\left(k\right)$, it is executed when the system is in steady state with regard to r(k). In other words, the identification occurs when $y_{2}^{\prime \prime }\left(k\right)$—driven by the setpoint r(k)—converges to a constant value ρ. In this case, considering the time window defined by $k_{s}<k<k_{u}$ in which $y_{2}^{\prime \prime }\left(k\right)$ is in its steady state, (3) can be rewritten as (4)—without initial conditions:

(4) $$\begin{array}{c} y^{\prime \prime }\left(k\right)=\underset{y_{1}^{\prime \prime }\left(k\right)}{\underset{︸}{w\left(k\right)*Z^{-1}\left[M\left(z\right)\left(\frac{1+C(z)P(z)}{1+C(z)P(z)M(z)}\right)\right]}}+\underset{y_{2}^{\prime \prime }\left(k\right)}{\underset{︸}{\rho }},\;\forall k_{s}<k<k_{u}, \end{array}$$

wherein ρ can be estimated by computing the average ${\bar{y}}^{\prime \prime }$ of $y^{\prime \prime }\left(k\right)$ during a certain amount of samples $\tau \leq (k_{u}-k_{s})$ starting at $k_{s}$, as indicated in (5):

(5) $$\begin{array}{c} {\bar{y}}^{\prime \prime }=\sum_{k_{s}}^{k_{s}+\tau }\frac{y^{\prime \prime }\left(k\right)}{\tau }=\underset{{\bar{y}}_{1}^{\prime \prime }\left(k\right)}{\underset{︸}{\sum_{k_{s}}^{k_{s}+\tau }\frac{w\left(k\right)*Z^{-1}\left[M\left(z\right)\left(\frac{1+C(z)P(z)}{1+C(z)P(z)M(z)}\right)\right]}{\tau }}}+\underset{{\bar{y}}_{2}^{\prime \prime }\left(k\right)}{\underset{︸}{\sum_{k_{s}}^{k_{s}+\tau }\frac{\rho }{\tau }}}, \end{array}$$

Considering that w(k)∼N(μ,σ), wherein μ=0 as previously stated, then ${\bar{y}}_{1}^{\prime \prime }\left(k\right)\to 0$ when τ→∞. In this case, for a sufficiently large τ, (5) can be simplified to (6):

(6) $$\begin{array}{c} {\bar{y}}^{\prime \prime }\approx \rho , \end{array}$$

Thus, by applying (6) in (4), we may define (7):

(7) $$\begin{array}{c} y_{1}^{\prime \prime }\left(k\right)\approx y^{\prime \prime }\left(k\right)-{\bar{y}}^{\prime \prime },\;\forall k_{s}<k<k_{u}, \end{array}$$

wherein $y_{1}^{\prime \prime }\left(k\right)$—obtained through measurements of $y^{\prime \prime }\left(k\right)$—is the output of the model defined by (8) when the noise w(k) is applied to its input:

(8) $$\begin{array}{c} y_{1}^{\prime \prime }\left(k\right)=w\left(k\right)*Z^{-1}\left[M\left(z\right)\left(\frac{1+C(z)P(z)}{1+C(z)P(z)M(z)}\right)\right]. \end{array}$$

Based on (8), if C(z) and P(z) are known, the Attack Identification process can estimate M(z) by applying w(k) in an estimated system, defined by (9):

(9) $$\begin{array}{c} {\hat{y}}_{1}^{\prime \prime }\left(k\right)=w\left(k\right)*Z^{-1}\left[M_{e}\left(z\right)\left(\frac{1+C(z)P(z)}{1+C\left(z\right)P\left(z\right)M_{e}\left(z\right)}\right)\right], \end{array}$$

wherein $M_{e}\left(z\right)$ is the estimation of M(z) and ${\hat{y}}_{1}^{\prime \prime }\left(k\right)$ is the output of the estimated system in face of $M_{e}\left(z\right)$. By comparing ${\hat{y}}_{1}^{\prime \prime }\left(k\right)$ with $y_{1}^{\prime \prime }\left(k\right)$, the Attack Identification process can evaluate whether $M_{e}\left(z\right)$ is equal/approximately M(z). Please note that $M_{e}\left(z\right)$ is a generic LTI attack function represented by (10):

(10) $$M_{e}\left(z\right)=\frac{\alpha_{n}z^{n}+\alpha_{n-1}z^{n-1}+\ldots +\alpha_{1}z^{1}+\alpha_{0}}{z^{m}+\beta_{m-1}z^{m-1}+\ldots +\beta_{1}z^{1}+\beta_{0}},$$

wherein *n* and *m* are the order of the numerator and denominator, respectively, while $\left[\alpha_{n},\alpha_{n-1},\ldots \alpha_{1},\alpha_{0}\right]$ and $[\beta_{m-1},$$\beta_{m-2},\ldots \beta_{1},\beta_{0}]$ are the coefficients of the numerator and denominator, respectively, that are intended to be found by Attack Identification algorithm. Therefore, to find M(z), the coefficients of $M_{e}\left(z\right)$ are adjusted until the estimated output ${\hat{y}}_{1}^{\prime \prime }\left(k\right)$ converges to $y_{1}^{\prime \prime }\left(k\right)$—obtained from measurements of $y^{\prime \prime }\left(k\right)$ in the real NCS.

In this work, the Backtracking Search Optimization algorithm (BSA) [43], is used to iteratively adjust the coefficients of $M_{e}\left(z\right)$, by minimizing a specific fitness function until $M_{e}\left(z\right)$ converges to the actual M(z). To compute the fitness of the BSA individuals, the noise w(k)—recorded while $y^{\prime \prime }\left(k\right)$ was being captured—is applied on the estimated system defined by (9) and (10), where the coefficients of $M_{e}\left(z\right)$ are the coordinates $x_{j}=[\alpha_{n,j},\alpha_{n-1,j},\ldots \alpha_{1,j},$$\alpha_{0,j},\beta_{m-1,j}$, $\beta_{m-2,j},\ldots \beta_{1,j},\beta_{0,j}]$ of an individual *j* of the BSA. Let ${\hat{y}}_{1j}^{\prime \prime }\left(k\right)$ be the output of the estimated model (9) (10) in face of w(k), when the coefficients of $M_{e}\left(z\right)$ are $x_{j}$. Then, the fitness $f_{j}$ of each individual *j* is obtained by comparing ${\hat{y}}_{1j}^{\prime \prime }\left(k\right)$ with $y_{1}^{\prime \prime }\left(k\right)$, according to (11):

(11) $$f_{j}=\frac{\sum_{k=0}^{N}{\left(y_{1}^{\prime \prime }\left(k\right)-{\hat{y}}_{1j}^{\prime \prime }\left(k\right)\right)}^{2}}{N},$$

wherein *N* is the number of samples that exist during a monitoring period *T* of $y_{1}^{\prime \prime }\left(k\right)$. Please note that $\min f_{j}$ occurs when $[\alpha_{n,j},\alpha_{n-1,j},\ldots \alpha_{1,j},\alpha_{0,j},$$\beta_{m-1,j},\beta_{m-2,j},\ldots$$\beta_{1,j},\beta_{0,j}]\to [\alpha_{n},\alpha_{n-1},\ldots \alpha_{1},\alpha_{0},\beta_{m-1},$$\beta_{m-2},\ldots \beta_{1},\beta_{0}]$, i.e., when the estimated $M_{e}\left(z\right)$ converges to M(z).

The attack identification process described in this section, without the use of the Noise Impulse Integration technique (to be described in Section 3.2), is summarized in Algorithm 1. 

**Algorithm 1:** Attack Identification without the NII technique.

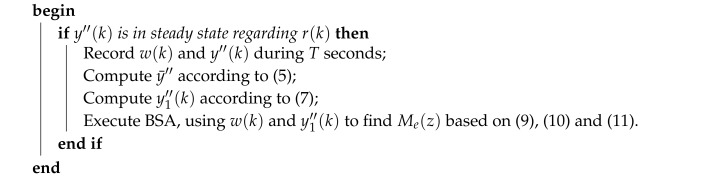



### 3.2. Integrating Impulses of Noise

This section presents the Noise Impulse Integration (NII) technique, which is added to the attack identification process described in Section 3.1 to improve its accuracy. This technique is inspired by the Pulse Integration process [42], used in pulse radar systems to improve the probability of detection and reduce the probability of false alarms in those systems. To allow a clear comprehension on the inspiration obtained from the radar Pulse Integration technique, it is necessary to provide a brief explanation on how a pulse radar system works and what is the main idea behind the pulse integration process. Therefore, first, Section 3.2.1 provides an explanation on the radar pulse integration process. Then, Section 3.2.2 introduces the NII technique.

#### 3.2.1. Radar Pulse Integration

**Figure 4:**
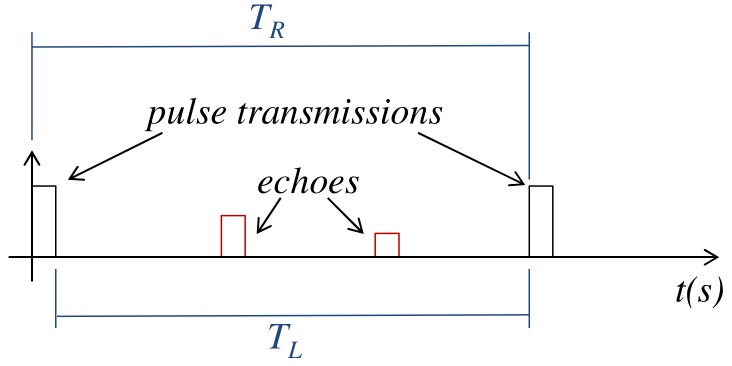
Pulse transmissions.

In a pulse radar system, the radar transmits electromagnetic pulses to the environment to detect and obtain information about targets. When a pulse reaches a reflective surface—of a target or other objects in the environment—it is reflected producing an echo that travels back to the radar antenna, allowing the target detection. To increase the probability of detection, the radar does not transmit only one pulse during the detection process. Instead, as depicted in Figure 4, the radar transmits a series of pulses, one at each pulse repetition interval $T_{R}$. Also, as shown in Figure 4, between two consecutive transmissions there is a silence period $T_{L}$ in which the radar remains listening the echoes that arrive from the monitored environment. These echoes may represent a target or another reflective body situated within the line of sight of the radar antenna.

**Figure 5:**
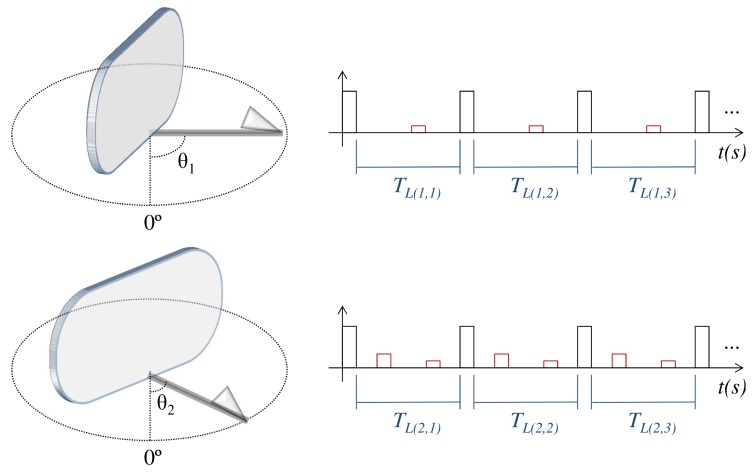
Radar scan process, in which a sequence of pulses is transmitted for each antenna pointing angle.

**Figure 6:**
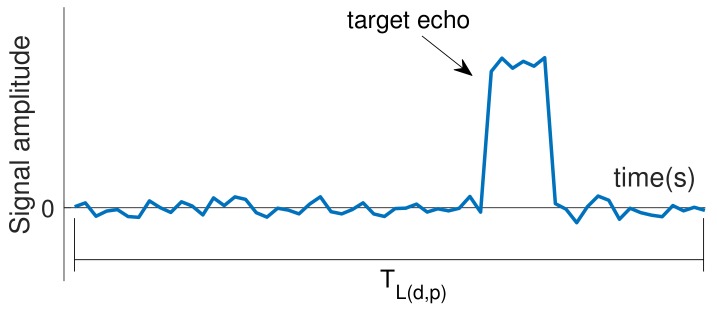
Noisy signal typically received during a given listening period $T_{L(d,p)}$.

Please note that while the radar scans the environment by rotating its antenna, for each antenna pointing angle θ, several pulses are transmitted in sequence as shown in Figure 5. Naturally, for each pulse *p* transmitted from a given antenna pointing angle $\theta_{d}$, there will be a listening period $T_{L(d,p)}$ to receive echoes. It happens that in a real system, the signal received during each listening period $T_{L(d,p)}$ does not contain only target echoes. Typically, as represented in Figure 6, the received signal also contains uncorrelated signal fluctuations (noise), whose amplitude follows a gaussian distribution with zero mean [47,48].

**Figure 7:**
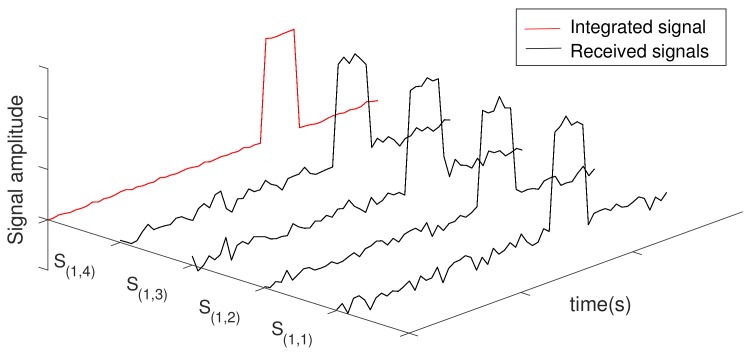
Radar pulse integration.

To increase signal-to-noise ratio (SNR), the radar Pulse Integration (RPI) technique combines the signals received in multiple listening periods $T_{L(d,p)}$ in a given $\theta_{d}$, taking advantage of the mentioned noise properties –i.e., uncorrelated fluctuations with gaussian distribution and zero mean. Basically, all signals $S_{\left(d,p\right)}\left(t\right)$ received in a sequence of listening periods $T_{L(d,p)}$ are integrated by computing their mean according to (12):

(12) $$I\left(t\right)=\frac{\sum_{p=1}^{h}S_{\left(d,p\right)}\left(t\right)}{h},$$

wherein I(t) is the integrated signal and *h* is the number of signals buffered in a sequence of listening periods. A representation of this computation is shown in Figure 7, where the signals received in a sequence of four listening periods (i.e., h=4) are buffered and integrated according to (12). Please note that the integrated signal has a better SNR when compared to the other signals. The uncorrelated noise is minimized (almost cancelled) thanks to its gaussian distribution with zero mean. On the other hand, the target echo (constantly present with non-zero mean amplitude) is reinforced. Ideally, the noise of the integrated signal is completely cancelled when h→∞. In this case, I(t) would contain only echoes.

#### 3.2.2. Noise Impulse Integration Technique

The NII technique described in this section works similarly to the RPI process described in Section 3.2.1. Basically, it integrates portions of noisy signals to cancel information that may disturb the identification process, and extract the information that is useful to obtain accurate models. Despite the inspiration obtained from the RPI, it is worth mentioning the following differences between both techniques:

- **Goal:** The goal of the RPI technique is to minimize the uncorrelated noise contained in signals received by the radar, and reinforce the echoes reflected by bodies within the radar antenna’s line of sight—i.e., produce a signal with grater SNR. The goal of the NII technique is to obtain a clear impulse response function of an LTI system, when it is excited by a white gaussian noise;
- **Integrated signals:** The RPI technique integrates signals received between consecutive pulse transmissions, containing, in general, reflected pulses and noise. The NII technique integrates portions of the signal produced by an LTI system when white gaussian noise is injected into it.
- **Selection of signals to be integrated:** In the RPI technique, the selection of signals to be integrated is straightforward. As explained in Section 3.2.1, it integrates signals received between the transmission of consecutive radar pulses. This selection provides a synchronism between the signals to be integrated, which, as shown in Figure 7, aligns the information that must be reinforced by the RPI—i.e., reinforce echoes that are constantly present in the received signal. The RPI’s signal selection cannot be used in the NII technique, given that the latter is not triggered by pulses. Therefore, it is necessary to use other criteria to select the portions of signal to be integrated, which is explained in the remainder of this section.

The white gaussian noise w(k), herein used to excite the LTI transfer function to be identified, can be defined as a sum of time-shifted impulses with uncorrelated random weights (amplitudes) as shown in (13):

(13) $$w\left(k\right)=\sum_{i=-\infty }^{\infty }\omega (i)\delta (k-i),$$

in which the amplitudes ω(i)∼N(μ,σ), *N* is a normal distribution, μ is its mean and σ is its non-zero standard deviation. When a weighted time-shifted impulse ω(i)δ(k−i) of w(k) is individually applied to a given LTI system H(z)=Z{h(k)}, it produces an output signal $y_{i}\left(k\right)$ defined by (14):

(14) $$\begin{array}{cc} y_{i}\left(k\right) & =\omega (i)\delta (k-i)*h(k)\\ \; & =\omega (i)h(k-i). \end{array}$$

Please note that $y_{i}\left(k\right)$ is the impulse response of h(k)—i.e., h(k) itself—weighted by the impulse’s amplitude ω(i) and time-shifted by *i* samples. However, when w(k) is applied to h(k), the output signal is no more composed by a single weighted time-shifted impulse response function. In this case, the discrete-time output y(k) produced when h(k) is excited by a white gaussian noise w(k) is determined by the discrete convolution (15):

(15) y(k)=w(k)∗h(k).

Considering (13), Equation (15) can be rewritten as (16) and (17):

(16) $$y\left(k\right)=\sum_{i=-\infty }^{\infty }\omega (i)\delta (k-i)*h\left(k\right)$$

(17) $$\begin{array}{c} y\left(k\right)=\sum_{i=-\infty }^{-1}\omega (i)\delta (k-i)*h\left(k\right)+\omega \left(0\right)\delta \left(k\right)*h\left(k\right)+\sum_{i=1}^{\infty }\omega (i)\delta (k-i)*h\left(k\right). \end{array}$$

which means that the output y(k) is composed by a sum of randomly weighted time-shifted impulse responses of h(k). Evidently, by observing (17), it is possible to verify that y(k) could result in a weighted impulse response of h(k) if conditions (18) and (19) were met:

(18) ω(0)≠0

(19) ω(i)=0,∀i≠0,

which would make it straightforward to reveal h(k) by measuring y(k). However, although condition (18) is possible, condition (19) is not feasible, given that ω(i)∼N(μ,σ), and σ≠0, as previously defined. Thus, the task of the NII technique is to overcome the constraint imposed by condition (19). Its goal is to produce a signal derived from y(k) that can reveal h(k) in the same way as if conditions (18) and (19) were met.

Inspired by the RPI, the NII technique consists of separating portions of y(k) that, when integrated, reinforce selected impulse responses of h(k) and minimize (cancel) the interferences produced by other weighted time-shifted impulse responses of h(k) contained in y(k). Therefore, let $y_{j}\left(k\right)$ be a portion of signal extracted from y(k), wherein *j* is a reference number used to identify each $y_{j}\left(k\right)$. The instances $y_{j}\left(k\right)$ are extracted from the output y(k) based on the amplitudes of the input signal w(k), which is evaluated during a monitoring period staring in sample $k_{f}$ and ending in sample $k_{l}$. This said, each $y_{j}\left(k\right)$ is obtained according Algorithm 2.

**Algorithm 2:** Generation of signals $y_{j}\left(k\right)$.

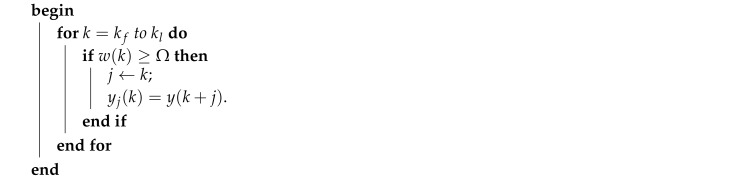



According to Algorithm 2, each *j* is a value of *k* in which the input w(k) is grater or equal than an amplitude threshold Ω. Please note that $y_{j}\left(k\right)$ is an instance of y(k) advanced (left-shifted) by *j* samples. Thus, in the same way that y(k) is defined by (17), $y_{j}\left(k\right)$ can be written as (20):

(20) $$\begin{array}{c} y_{j}\left(k\right)=\sum_{i=-\infty }^{-1}\omega_{j}\left(i\right)\delta \left(k-i\right)*h\left(k\right)+\omega_{j}\left(0\right)\delta \left(k\right)*h\left(k\right)+\sum_{i=1}^{\infty }\omega_{j}\left(i\right)\delta \left(k-i\right)*h\left(k\right) \end{array}$$

wherein $\omega_{j}\left(i\right)$, defined according to (21), are the advanced (left-shifted) amplitudes of the white gaussian noise (13):

(21) $$\omega_{j}\left(i\right)=\omega \left(i+j\right).$$

Considering that Algorithm 2 is intended to produce a collection of $y_{j}\left(k\right)$—which is necessary for the NII technique—let *J* be the set of all *j*, and |J| be the total number of elements j∈J. Therefore, analogously to the RPI process, the mean Υ(k) of all $y_{j}\left(k\right)$ is computed according to (22):

(22) $$\Upsilon \left(k\right)=\frac{\sum_{j\in J}y_{j}\left(k\right)}{\left|J\right|},$$

Thus, considering (20), Equation (22) can be rewritten as (23):

(23) $$\begin{array}{c} \Upsilon \left(k\right)=\underset{\Upsilon_{1}\left(k\right)}{\underset{︸}{\frac{\sum_{j\in J}\left[\sum_{i=-\infty }^{-1}\omega_{j}\left(i\right)\delta \left(k-i\right)*h\left(k\right)\right]}{\left|J\right|}}}+\underset{\Upsilon_{2}\left(k\right)}{\underset{︸}{\frac{\sum_{j\in J}\omega_{j}\left(0\right)\delta \left(k\right)*h\left(k\right)}{\left|J\right|}}}+\underset{\Upsilon_{3}\left(k\right)}{\underset{︸}{\frac{\sum_{j\in J}\left[\sum_{i=1}^{\infty }\omega_{j}\left(i\right)\delta \left(k-i\right)*h\left(k\right)\right]}{\left|J\right|}}} \end{array}$$

Please note that $\omega_{j}\left(i\right)$ has the same probability distribution function of ω(i) (i.e., $\omega_{j}\left(i\right)∼N\left(\mu ,\sigma \right)$) since, according to (21), $\omega_{j}\left(i\right)$ consists of the same amplitudes of ω(i), but left-shifted. Thus, considering that μ=0, then $\Upsilon_{1}\left(k\right)\to 0$ and $\Upsilon_{3}\left(k\right)\to 0$ when |J| increases. It means that for a given i≠0 the impulse responses produced by all $\omega_{j}\left(i\right)\delta \left(k-i\right)$ are canceled when the average of $y_{j}\left(k\right)$ is computed among all j∈J.

On the other hand, $\Upsilon_{2}\left(k\right)\neq 0$ since that the mean of $\omega_{j}\left(0\right)$, among all j∈J, is different from zero. Please note that according to (21) $\omega_{j}\left(0\right)=\omega \left(j\right)$. From Algorithm 2, w(j)≥Ω which, according to (13), means that ω(j)≥Ω. Therefore, $\omega_{j}\left(0\right)\geq \Omega$, ∀j. This reasoning demonstrates that the mean of all $\omega_{j}\left(0\right)$ is greater than Ω and, therefore, $\Upsilon_{2}\left(k\right)\neq 0$. In this case, the responses produced by all $\omega_{j}\left(0\right)\delta \left(k\right)$ are the impulses responses of h(k) selected to be reinforced through the NII technique. This reinforcement is analogous to what the RPI technique does with target echoes. This said, (23) can be simplified as (24):

(24) $$\Upsilon \left(k\right)={\bar{\omega }}_{j}\left(0\right)\delta \left(k\right)*h\left(k\right),$$

wherein ${\bar{\omega }}_{j}\left(0\right)$ is the mean of all $\omega_{j}\left(0\right)$, according to (25):

(25) $${\bar{\omega }}_{j}\left(0\right)=\frac{\sum_{j\in J}\omega_{j}\left(0\right)}{\left|J\right|}.$$

**Figure 8:**
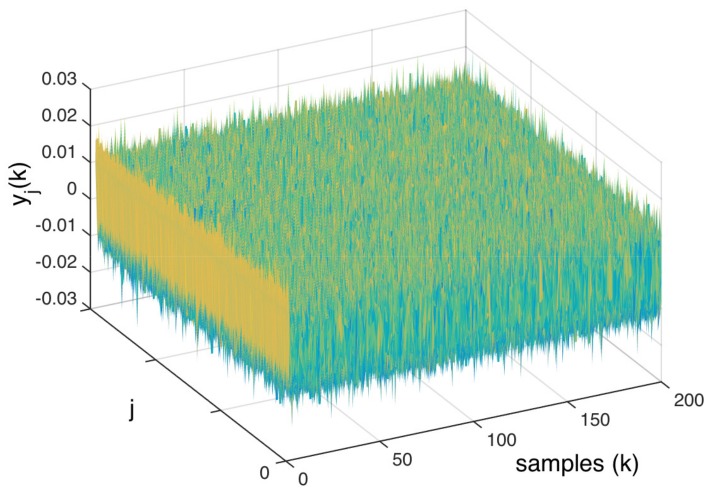
Signals $y_{j}\left(k\right)$ aligned to be integrated.

**Figure 9:**
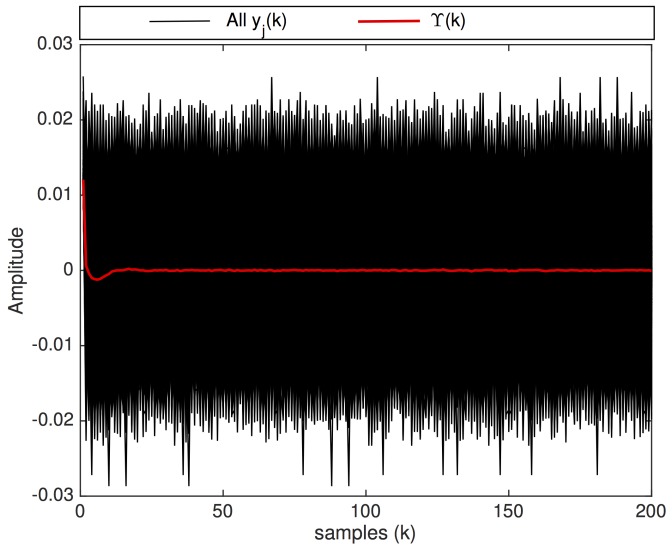
The impulse response Υ(k) (in red) produced by the NII technique after the integration of a set of signals $y_{j}\left(k\right)$ (shown overlapped in black).

An example of the computation performed by the NII technique is represented in Figure 8 and Figure 9. In this example, the transfer function H(z) (26):

(26) $$H\left(z\right)=Z\left[h\left(k\right)\right]=\frac{z^{3}-2.546z^{2}+2.111z-0.5646}{z^{3}-2.489z^{2}+2.102z-0.6113},$$

is excited by the white gaussian noise w(k) (13), with ω(i)∼N(0,0.005), thereby providing the output y(k)=w(k)∗h(k). The amplitude threshold Ω of Algorithm 2, used to obtain all $y_{j}\left(k\right)$ from y(k), is Ω=0.01. Figure 8 shows all $y_{j}\left(k\right)$ aligned side by side to be integrated (similarly to the representation shown in Figure 7 for the RPI process). This figure—when compared to Figure 7—depicts the analogy between the NII and the RPI techniques, showing signals whose uncorrelated noise can be cancelled through (22) in order to obtain the desired information—which here, in the NII technique, is the weighted impulse response Υ(k) of H(z).

Figure 9 shows the signal Υ(k) produced by the computation of (22) using the set of signals represented in Figure 8. The signal Υ(k), highlighted in red, is the result of the integration of all $y_{j}\left(k\right)$ which, according to (24), reveals the impulse response of the system as it was excited by the impulse ${\bar{\omega }}_{j}\left(0\right)\delta \left(k\right)$. To graphically compare the magnitude of Υ(k) with the noise magnitude, Figure 9 also shows all $y_{j}\left(k\right)$ of overlapped in black—as a front view of Figure 8. Therefore, in Figure 9, Υ(k) (in red) is the signal of interest, which is extracted from all noisy signals $y_{j}\left(k\right)$ (overlapped in black) when the uncorrelated noise of all $y_{j}\left(k\right)$ is cancelled by computing (22).

As previously discussed, the NII technique is herein used to complement the attack identification strategy described in Section 3.1 to improve its accuracy. To do so, let us consider that:

- ${\bar{\omega }}_{j}\left(0\right)$ and Υ(k) are obtained through the NII technique, by processing signals w(k) and $y_{1}^{\prime \prime }\left(k\right)$—specified in Section 3.1 and indicated in Figure 3;
- h(k) is the transfer function between w(k) and $y_{1}^{\prime \prime }\left(k\right)$ which, according to (8), is defined as (27):
  (27) $$\begin{array}{c} h\left(k\right)=Z^{-1}\left[M\left(z\right)\left(\frac{1+C(z)P(z)}{1+C(z)P(z)M(z)}\right)\right]. \end{array}$$

Doing so, (24) can be rewritten as (28):

(28) $$\begin{array}{c} \Upsilon \left(k\right)={\bar{\omega }}_{j}\left(0\right)\delta \left(k\right)*Z^{-1}\left[M\left(z\right)\left(\frac{1+C(z)P(z)}{1+C(z)P(z)M(z)}\right)\right], \end{array}$$

which can now be used to estimate M(z) in the same way as in Section 3.1 for equation (8). Please note that the differences between (8) and (28) are:

- the input of (8) is a white gaussian noise and its output is a white gaussian noise filtered by h(k);
- the input of (28) is a weighted impulse signal and its output is a weighted impulse response of h(k).

Now, given (28), the attack function M(z) can be estimated by an optimization algorithm (e.g., the BSA), such as described in Section 3.1. In this case, if C(z) and P(z) are known (which is feasible for the NCS owner), M(z) can be estimated by applying ${\bar{\omega }}_{j}\left(0\right)\delta \left(k\right)$ in an estimated system, defined by (29):

(29) $$\begin{array}{c} \hat{\Upsilon }\left(k\right)={\bar{\omega }}_{j}\left(0\right)\delta \left(k\right)*Z^{-1}\left[M_{e}\left(z\right)\left(\frac{1+C(z)P(z)}{1+C\left(z\right)P\left(z\right)M_{e}\left(z\right)}\right)\right], \end{array}$$

wherein $M_{e}\left(z\right)$ is the estimation of M(z) and $\hat{\Upsilon }\left(k\right)$ is the output of the estimated system in face of $M_{e}\left(z\right)$. Recall that $M_{e}\left(z\right)$ is the generic LTI attack function represented by (10) wherein $\left[\alpha_{n},\alpha_{n-1},\ldots \alpha_{1},\alpha_{0}\right]$ and $[\beta_{m-1},$$\beta_{m-2},\ldots \beta_{1},\beta_{0}]$ are the coefficients of the numerator and denominator, respectively, that are intended to be found by Attack Identification algorithm. By comparing $\hat{\Upsilon }\left(k\right)$ with Υ(k), the Attack Identification process can evaluate whether $M_{e}\left(z\right)$ is equal to/approximately M(z).

In the same way as in Section 3.1, to discover M(z), the coefficients of $M_{e}\left(z\right)$ are adjusted by the BSA until the estimated output $\hat{\Upsilon }\left(k\right)$ converges to Υ(k) (the latter obtained by the NII technique from measurements of $y^{\prime \prime }\left(k\right)$ and w(k) in the real NCS). Let $\hat{\Upsilon }j\left(k\right)$ be the output of the estimated model (29) in face of the input ${\bar{\omega }}_{j}\left(0\right)\delta \left(k\right)$, when the coefficients of $M_{e}\left(z\right)$ (10) are the coordinates $x_{j}=[\alpha_{n,j},\alpha_{n-1,j},\ldots \alpha_{1,j},$$\alpha_{0,j},\beta_{m-1,j}$, $\beta_{m-2,j},\ldots \beta_{1,j},\beta_{0,j}]$ of an individual *j* of the BSA. In this case, the fitness $f_{j}$ of each individual *j* of the BSA is obtained comparing $\hat{\Upsilon }j\left(k\right)$ with Υ(k), according to (30):

(30) $$f_{j}=\frac{\sum_{k=0}^{N}{\left(\Upsilon \left(k\right)-\hat{\Upsilon }j\left(k\right)\right)}^{2}}{N},$$

wherein N is the number of samples that exist in Υ(k). As already discussed in Section 3.1, $\min f_{j}$ occurs when $[\alpha_{n,j},\alpha_{n-1,j},\ldots \alpha_{1,j},\alpha_{0,j},$$\beta_{m-1,j},\beta_{m-2,j},\ldots$$\beta_{1,j},\beta_{0,j}]\to [\alpha_{n},\alpha_{n-1},\ldots \alpha_{1},\alpha_{0},\beta_{m-1},$$\beta_{m-2},\ldots \beta_{1},\beta_{0}]$, i.e., when the estimated $M_{e}\left(z\right)$ converges to M(z).

The complete attack identification process described in this section, performed with the Noise Impulse Integration technique, is summarized in Algorithm 3. Please note that the differences between Algorithms 1 and 3 is that the former does not have the NII stage. This way, while Algorithm 3 uses w(k) and $y_{1}^{\prime \prime }\left(k\right)$ as input signals to the BSA-based identification, Algorithm 3 uses ${\bar{\omega }}_{j}\left(0\right)\delta \left(k\right)$ and Υ(k) as input signals to the BSA-based identification.

**Algorithm 3:** Attack Identification with the NII technique.

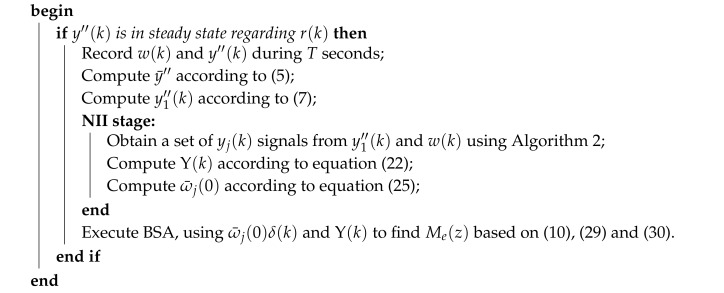



## 4. Results

This section analyses the performance of the attack identification strategy proposed in Section 3 when identifying the Controlled Data Injection attack characterized in Section 2. The evaluation on the accuracy of the countermeasure is based on results obtained through simulations using MATLAB/SIMULINK. First, Section 4.1 describes the attacked NCS and the attack parameters. Then, Section 4.2 presents the results obtained by the proposed countermeasure in the scenario described in Section 4.1.

### 4.1. Attacked NCSs and Parameters of the Attack

In the simulations of this section, the attacked NCS has the same architecture of the NCS shown in Figure 3. The system consists of Proportional-Integral (PI) controller that controls the rotational speed of a DC motor—which has broad applications in industry and real-world systems, and has been widely used in previous works about NCS [8,49,50,51,52]. The control function C(z) and the plant transfer function P(z) are the same as in [8,50], which are represented by (31):

(31) $$C\left(z\right)=\frac{0.1701z-0.1673}{z-1}\;P\left(z\right)=\frac{0.3379z+0.2793}{z^{2}-1.5462z+0.5646}$$

The sample rate of the system is 50 samples/s and the set point r(k) is a unitary step function.

As discussed in [8], one way to degrade the service of a plant is by causing overshoots during its transient response, which, indeed, can cause stress and possibly damage a variety of physical systems [53]. Thus, in this work, an attack function M(z) is designed to degrade the plant service by causing 50% of overshoot in the motor speed. To achieve this goal, a MitM located in the feedback link runs the attack function represented by (32), wherein $\alpha_{0}=0.25$ and $\beta_{0}=-0.75$:

(32) $$M\left(z\right)=\frac{\alpha_{0}}{z+\beta_{0}}.$$

### 4.2. Performance of the Attack Identification

Section 3 proposes an attack identification process where the NII technique is used to improve the accuracy of the estimation of LTI attack functions in NCSs. This section analyzes the performance of the proposed attack identification method when estimating the attack defined in Section 4.1. To statistically evaluate how the NII technique improves the accuracy of the identification process, two set of simulations are carried out:

1. 100 simulations using the identification process shown in Algorithm 1—i.e., without the NII technique; and
2. 100 simulations using the identification process shown in Algorithm 3—i.e., with the NII technique.

The noise w(k)∼N(μ,σ) injected in the system by the identification scheme is configured with μ=0 and σ=0.005, which makes 95% of the noise amplitudes within ±0.01 (these parameters are chosen to produce a small noise, considering the magnitude of the plant output signal transmitted through the feedback link). Each of the 100 simulations with Algorithms 1 and 3 uses a different (randomly generated) white gaussian noise signal.

**Figure 10:**
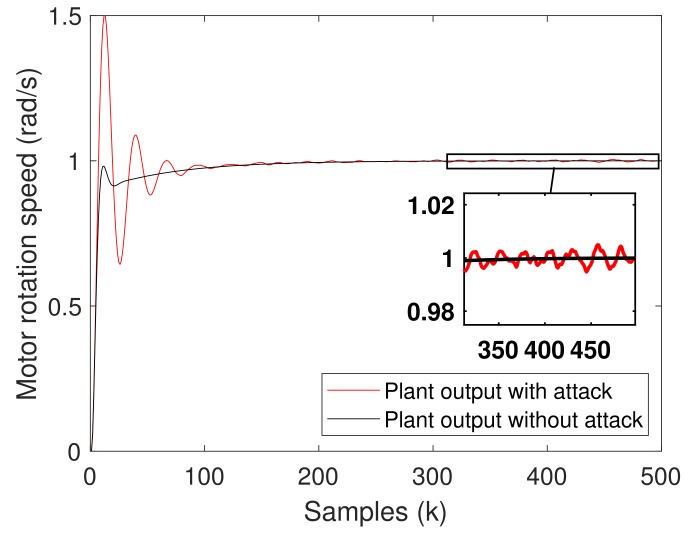
Motor speed with and without attack.

Figure 10 shows examples of the system output (the motor speed) with and without the attack. Please note that when the attack is executed, the motor speed has an overshoot of 50% and a small noise is present in the plant output. However, in a normal condition—i.e., without attack—the noise is cancelled and does not appear in the plant output (as expected, based on Equation (1) when M(z)=1).

As previously discussed, the present attack identification scheme aims to estimate the coefficients of M(z), which according to (32) are $\alpha_{0}$ and $\beta_{0}$. The BSA settings in both Algorithms 1 and 3 are the same as those used in [8,45]: the lower and upper limits of each search space dimension are −10 and 10, respectively; the BSA population has 100 individuals; and η=1 (in the BSA, η is used to define the amplitude of the displacement of the individuals).

The BSA is executed for 600 iterations.

For the execution of Algorithm 1 the signals w(k) and $y^{\prime \prime }\left(k\right)$ are recorded during 100 samples, starting when the system achieves its steady state regarding to r(k). Thus, the size of signals w(k) and $y_{1}^{\prime \prime }\left(k\right)$ used by the BSA in (9) and (11), respectively, is N=100 samples. For the execution of Algorithm 3 the signals w(k) and $y^{\prime \prime }\left(k\right)$ are recorded during 0,5Msamples, also starting when the system achieves its steady state regarding to r(k). Recall that in Algorithm 3, the recorded signals are not directly applied to the BSA process. They are processed through the NII stage to result in ${\bar{\omega }}_{j}\left(0\right)\delta \left(k\right)$ and Υ(k). The signals ${\bar{\omega }}_{j}\left(0\right)\delta \left(k\right)$ and Υ(k) used by the BSA in (29) and (30), respectively, are sized with N=100 samples. This way, the signals processed by the BSA have the same size in both Algorithms 1 and 3 (i.e., N=N). The amplitude threshold of the NII is Ω=0.01, which means that the condition defined in Algorithm 2 (i.e., w(k)≥Ω) is true in approximately 2.28% of the samples of w(k).

**Figure 11:**
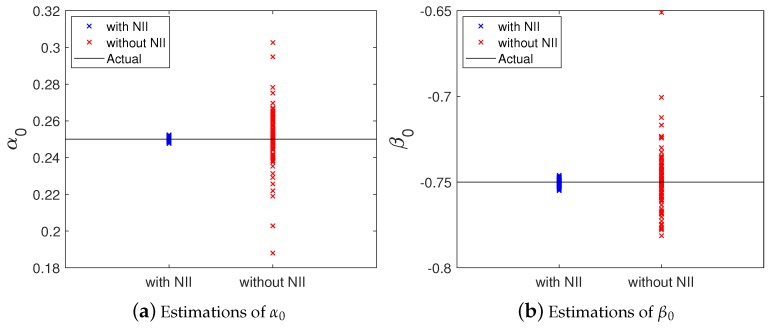
Estimations of $\alpha_{0}$ and $\beta_{0}$ with and without the NII stage. (**a**) Estimations of $\alpha_{0}$. (**b**) Estimations of $\beta_{0}$

Figure 11 shows the 100 values of $\alpha_{0}$ and $\beta_{0}$ estimated by the identification processes with and without the NII stage (i.e., with Algorithms 1 and 3, respectively). Additionally, Table 1 shows the statistics of the results presented in Figure 11. From Figure 11 and Table 1, it is possible to verify that the accuracy of the attack identification algorithm with the NII stage is better than the accuracy obtained without the proposed technique. Figure 11 demonstrates that with the NII stage, the estimated values of $\alpha_{0}$ and $\beta_{0}$ are closer to their actual values—i.e., less spread—than without the NII stage. Please note that the statistics shown in Table 1 ratifies the better performance provided by the NII stage. In this case, the means of the estimated values are closer to the to the real values of $\alpha_{0}$ and $\beta_{0}$, with lower standard deviation.

**Figure 12:**
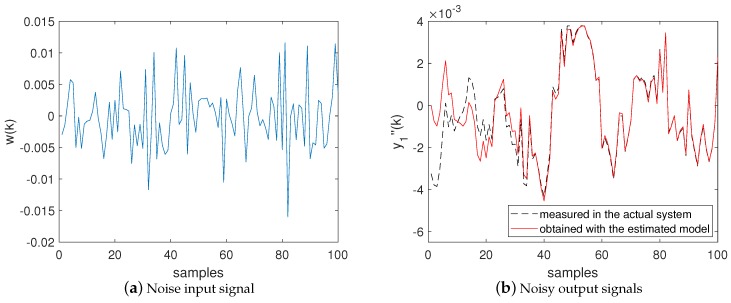
Input and output signals used by the BSA in Algorithm 1 to estimate M(z) considering the model defined in (8). (**a**) Noise input signal. (**b**) Noisy output signals.

Figure 12 shows the input and output signals used by the BSA to estimate M(z) in a simulation example performed with Algorithm 1 (without the NII stage). Figure 12a shows the noise w(k) recorded in the actual system and used by the BSA as input for the model defined by (8). Figure 12b shows in black dashed line the signal $y_{1}^{\prime \prime }\left(k\right)$ measured in the actual system and used by the BSA as the reference output for the model defined by (8). Additionally, Figure 12b shows in red line the signal ${\hat{y}}_{1}^{\prime \prime }\left(k\right)$ produced by the estimated model—i.e., the model (9) containing the estimated attack function—when excited by the noise input shown in Figure 12a. In Figure 12b, it is possible to see that the output ${\hat{y}}_{1}^{\prime \prime }\left(k\right)$ obtained with the estimated model does not completely match the output $y_{1}^{\prime \prime }\left(k\right)$ measured in the actual system. It exemplifies, as shown in Figure 11 and Table 1, the lower accuracy of Algorithm 1 when identifying M(z).

**Figure 13:**
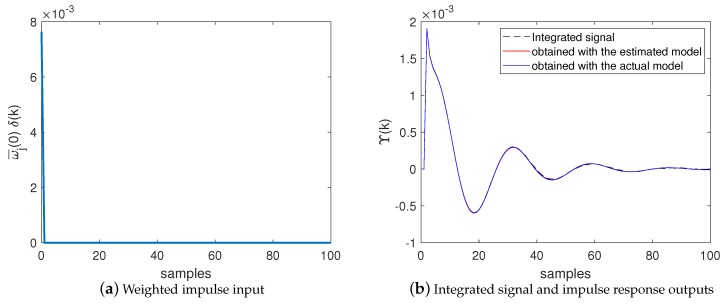
Input and output signals used by the BSA in Algorithm 3 to estimate M(z) considering the model defined in (28). (**a**) Weighted impulse input. (**b**) Integrated signal and impulse response outputs.

Figure 13, in turn, shows the input and output signals used by the BSA to estimate M(z) in a simulation example performed with Algorithm 3 (with the NII stage). Figure 13a shows the weighted impulse ${\bar{\omega }}_{j}\left(0\right)\delta \left(k\right)$ produced by the NII stage and used by the BSA as input for the model defined in (28). Figure 13b shows:

- In black dashed line: the integrated signal Υ(k) produced by the NII stage (based on measurements in the actual system) and used by the BSA as the reference output for the model defined in (28);
- In blue line: the impulse response produced when the weighted impulse ${\bar{\omega }}_{j}\left(0\right)\delta \left(k\right)$, shown Figure 13a, is applied to the system defined in (28) containing the actual attack function;
- In red line: the impulse response produced when the weighted impulse ${\bar{\omega }}_{j}\left(0\right)\delta \left(k\right)$, shown Figure 13a, is applied to the system defined in (29) containing the estimated attack function.

From Figure 13b, it is possible to see that the integrated signal (provided by the NII stage) accurately meets the impulse response of the actual system. It indicates that the NII technique can accurately reveal the impulse response of the system based on the signals produced by the white gaussian noise injected in the NCS. Additionally, Figure 13b shows that the impulse response obtained with the estimated model accurately meets the impulse response obtained with the actual system. It demonstrates that NII stage effectively contributes to enhance the accuracy of the identification process, as already shown in Figure 11 and Table 1.

The better performance obtained with the NII stage is mainly attributed to the cancelation of the initial conditions produced by the noise in the actual system. Please note that in Algorithm 1, the noise input was already present in the system since before $y_{1}^{\prime \prime }\left(k\right)$ was obtained, which makes w(k) affect the initial conditions of the system. Thus, the lack of knowledge about the initial conditions of the system affects the estimation of the attack function in Algorithm 1. On the other hand, in Algorithm 3, the impact of w(k) in the system’s initial conditions is mitigated by the NII stage. This statement can be verified in Equation (23), where $\Upsilon_{1}\left(k\right)\to 0$ when all $y_{j}\left(k\right)$ are integrated among all j∈J, as demonstrated in Section 3.2.2. Indeed, when the noise input w(k) is transformed into a weighted impulse signal ${\bar{\omega }}_{j}\left(0\right)\delta \left(k\right)$, it is not expected to exist any initial conditions caused by w(k) in the system defined in (28), given that ${\bar{\omega }}_{j}\left(0\right)\delta \left(k\right)=0$, ∀−∞≤k<0.

**Figure 14:**
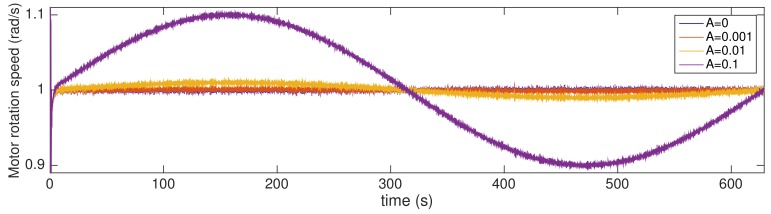
Examples of sensor outputs *y*′(*k*) in simulations with different amplitudes *A*.

Additionally, the performance of the proposed countermeasure is evaluated in scenarios where the reference signal is slowly changing during the execution of Algorithm 3. For this purpose, the reference signal of the system described in Section 4.1 is changed to (33):

(33) $$r\left(k\right)=\left\{\begin{array}{cc} 0 & \;\;\;\;k<0;\\ 1+A\;sin(0.01k) & \;\;\;\;k\geq 0. \end{array}\right.$$

where three different amplitudes *A* are considered: 0.001, 0.01, and 0.1 (i.e., 0.1%, 1% and 10% of the unitary step function setpoint, respectively). Please note that the reference signal of the original system described in Section 4.1 corresponds to the case where A=0. For statistical analysis, 100 different simulations are provided for each amplitude *A*. Figure 14 shows examples of output signals $y^{\prime }\left(k\right)$ obtained in simulations using different amplitudes *A* in (33).

**Figure 15:**
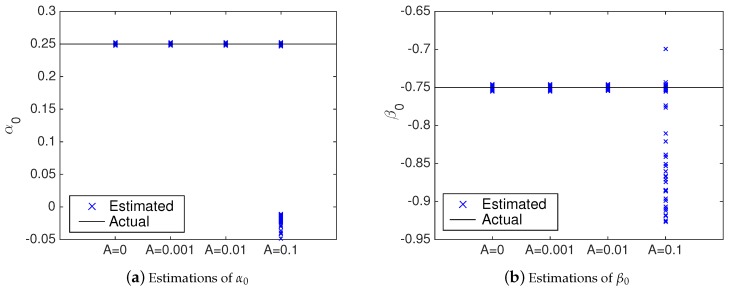
Estimations of $\alpha_{0}$ and $\beta_{0}$ using the NII technique, with and without variations in the reference signal r(k).

Figure 15 compares the coefficients estimated by Algorithm 3 when the reference signal is constant (A=0) and when it slowly varies using different amplitude values *A*. Additionally, Table 2 shows the statistics of the results presented in Figure 15. From Figure 15 and Table 2, it is possible to verify that the accuracy of the attack identification algorithm is not affected by variations in the reference signal when A=0.001 and A=0.01. In these cases, as shown in Figure 15, the estimated coefficients are quite close to their actual values and are as accurate as when A=0 (when there are no variations in r(k)). The statistics shown in Table 2 ratifies that for A=0.001 and A=0.01, the algorithm presents the same performance as when r(k) is not varying—the means and the standard deviations are practically the same as when A=0.

Lower performance is verified when the amplitude is increased to A=0.1. In this case, 29% of the estimated coefficients have their accuracy affected—these outliers can be seen in Figure 15 (specially in Figure 15a) far from the coefficients’ actual values. According to Table 2, when these outliers are taken into account, the means of the estimated coefficients diverge from the actual values and the standard deviations increase. However, it is worth mentioning that when A=0.1, even with the reduced performance, 71% of the results are not affected by the variations in r(k) and the estimated coefficients are as accurate as when A=0. Figure 15 shows these 71% of estimated coefficients close to their actual values. Moreover, Table 2 shows that if the outliers are not taken into account, the means and the standard deviations are practically the same as when A=0. These results suggest that even when the reference signal is (slowly) varying ±10%, the proposed algorithm can provide satisfactory performance in most cases (71%).

**Figure 16:**
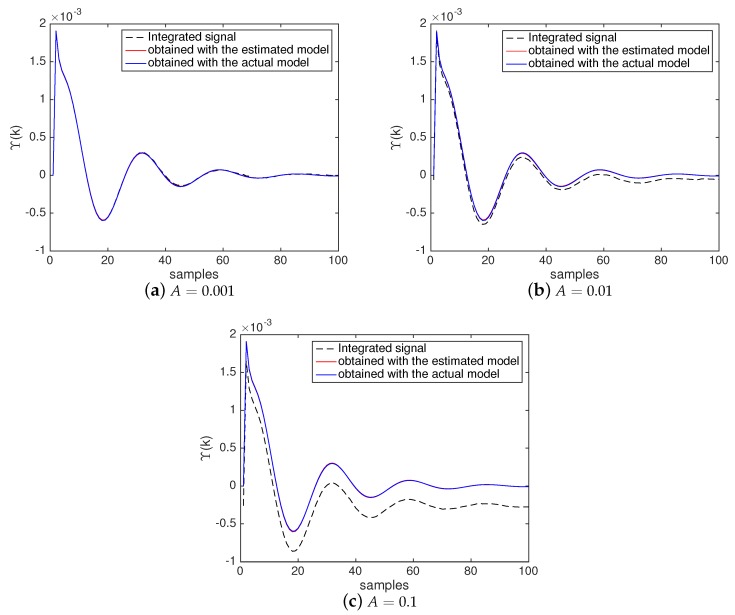
Examples of integrated signals and weighted impulse responses obtained through Algorithm 3 in scenarios with different amplitudes *A*. (**a**) *A* = 0.001; (**b**) *A* = 0.01; (**c**) A = 0.1.

For the sake of comparison with Figure 13b, Figure 16 brings examples of results obtained in scenarios with different amplitudes *A*. Through these examples, it is possible to visualize the impact of the different amplitudes *A* in the integrated signal Υ(k) produced by the NII stage, and in the weighted impulse response of the system containing the estimated attack function.

Note in Figure 16 that even with variations in the reference signal, the NII technique is able to accurately reveal the impulse response of the system under attack (represented in black dashed line). However, it is possible to see the presence of an offset between the integrated signal and the impulse response of the actual system. This offset tends to increase as *A* becomes higher, and is caused by the different levels of $y^{\prime \prime }\left(k\right)$ (due the variations in r(k)) at the time when w(k)≥Ω is satisfied and each $y_{j}\left(k\right)$ is obtained according to Algorithm 2. Still, even when such offset is higher (as in Figure 16c, when A=0.1), the optimization metaheuristic in most cases (71%) is able to accurately find the coefficients of M(z) by minimizing the difference between the impulse response of the system with the estimated model (represented in red) and the integrated signal (represented in black dashed line), making them parallel. In all examples shown in Figure 16, the impulse response of the system with the estimated model (represented in red) converges to the impulse response of the system with the actual model (represented in blue), illustrating the good accuracy obtained by the countermeasure even when r(k) is varying.

The results of this section indicate the effectiveness and accuracy of the proposed countermeasure when identifying SD-Controlled Data Injection attacks in NCSs, especially when the NII technique is used. The performance of the countermeasure, designed for scenarios where the reference signal remains constant, is evaluated considering r(k) as a step function. Additionally, the countermeasure is also evaluated in scenarios where the reference signal slowly changes, causing oscillations in the monitored signals (which in the present work are sensor measurements). In both cases, accurate results are obtained, with a performance reduction when the oscillation amplitude is increased to A=0.1. The results indicate that if the offsets shown in Figure 16 (introduced by variations in r(k)) are reduced, or taken into account in the BSA optimization process, the performance of the countermeasure can be further improved for reference signals varying with higher amplitudes—encouraging research in this direction.

Moreover, it is worth mentioning that in a normal conditions, when the system is not under attack, the injected noise is cancelled and does not affect the NCS. When the system is under attack, it is possible to see that noise is present in the plant output, but it is small due the parameters chosen for w(k). It should be noted that such small noise is not necessarily a drawback for the system; however, the possible impacts of this noise in case of attack have to be evaluated for each specific system.

## 5. Conclusions

This paper proposes a BSA-based countermeasure to identify LTI attack functions executed during SD-Controlled Data Injection attacks in NCSs. It consists of a link monitoring strategy that uses white gaussian noise to excite the attack function and, thus, produce signals with the information necessary for the identification process. The proposed solution is evaluated through simulations where the attacker aims to manipulate the measurements transmitted by the plant sensor. It is demonstrated that in normal operating conditions—i.e., without attack—the injected white gaussian noise is cancelled and does not affect the plant output. The injected white gaussian noise only manifests itself in the plant when an attack is occurring. In this case, the presence of noise may not necessarily be a drawback, but the possible impacts of such noise (in case of attack) must be evaluated according to the requirements of each specific plant.

Additionally, to increase the accuracy of the proposed countermeasure, this paper introduces the NII technique which is developed using the radar pulse integration process as inspiration. It is proven that the NII technique can accurately reveal the impulse response of the system under attack based on the signals produced by the white gaussian noise injected in the NCS. The results indicate that the NII technique indeed increases the accuracy of the attack identification, eliminating the need to estimate the initial conditions caused by the noise injected into the NCS.

Although the proposed countermeasure is designed for scenarios where the reference signal remains constant, we also evaluate it in scenarios where the reference signal slowly changes. In both cases, accurate results can be obtained, with a performance reduction when the oscillation of the reference signal increases. With this regard, the results indicate that if the integrated signal offset (introduced by variations in r(k)) is reduced, or taken into account in the BSA optimization process, the performance of the countermeasure can be further improved for reference signals varying with higher amplitudes. Therefore, we encourage future research in this direction.

As future work we plan to investigate the possibility of generalizing the proposed countermeasure, to be able to run the identification task when the system is either in steady or transient operating conditions. Moreover, we plan to evaluate the performance of the proposed countermeasure in identifying switching LTI attack functions.

Also, we consider to investigate the use of the NII technique as an attack tool for System Identification attacks [8,13] in scenarios with high data loss. In this paper, the technique is used to enhance the performance of a countermeasure in scenarios where the monitored signals are not impaired by data loss. However, we consider that the NII technique may be a useful tool to rebuild and reveal the impulse response functions of LTI systems in scenarios where the captured data is impaired by high percentage of loss. Such ability can be used, for instance, to enhance System Identifications attacks [8,13] in scenarios with extreme data loss—as in the case of an attacker far from WNCS transmitters, with poor connectivity, trying to identify the WNCS models.

## Figures and Tables

**Table 1:** Statistics of the attack identification process.

Coefficient	Algorithm	Mean	Standard Deviation
$\alpha_{0}$	with NII	0.2500	0.0011
	without NII	0.2506	0.0147
$\beta_{0}$	with NII	−0.7502	0.0017
	without NII	−0.7485	0.0172

**Table 2:** Statistics of the attack identification process using the NII technique, with and without variations in the reference signal r(k).

Coefficient	A	Mean	Standard Deviation
$\alpha_{0}$	0 *	0.2500	0.0011
	0.001	0.2500	0.0011
	0.01	0.2500	0.0011
	0.1	0.1712	0.1241
	0.1 **	0.2500	0.0012
$\beta_{0}$	0 *	−0.7502	0.0017
	0.001	−0.7502	0.0017
	0.01	−0.7501	0.0017
	0.1	−0.7812	0.0586
	0.1 **	−0.7500	0.0020

${}^{*}$ The reference signal r(k) does not vary. ${}^{**}$ Not taking into account the outliers verified in Figure 15. (These statistics represent the remaining 71% of the results.
